# Letter to editor regarding: “Spontaneous spinal epidural abscess in an adolescent patient: A case report and literature review”

**DOI:** 10.1016/j.ijscr.2023.108994

**Published:** 2023-10-28

**Authors:** Borislav Kitov, Atanas N. Davarski, Tanya Kitova

**Affiliations:** a Clinic of Neurosurgery, St. George University Hospital, Plovdiv, Bulgaria; b Department of Neurosurgery, Faculty of Medicine, Medical University of Plovdiv, Plovdiv, Bulgaria; c Department of Anatomy, Histology and Embryology, Medical Faculty, Medical University of Plovdiv, Plovdiv, Bulgaria*;* Medical College, Trakia University, Stara Zagora, Bulgaria

**Keywords:** Lumbar back pain, MRI, Epidural abscess, Spine

## Dear Editor,

It was with great interest that we read the article by Lim and Jo published in your esteemed journal [1]. There is no doubt that a spontaneous spinal epidural abscess (SSE) is a rare disease in adolescence, creating a challenge for clinicians. In this regard, Lim and Jo's submission is extremely valuable.

After a careful study of the article, we have some considerations that we would like to share with the readers of your journal. There are two types of SSEA: a/ primary (from a remote focal infection by a hematogenous route (arterial or venous) or through lymphatic vessels directly into the epidural space) and b/ secondary – per continuitatem from an adjacent infected structure (spondylodiscitis, septic arthritis of a facet joint or paravertebral abscess) [2]. In the presented Fig. 1. we are left with the impression that this is a case of secondary SSEA, which arose from a right sided septic arthritis of the facet joint (SAFS) on the L_4_-L_5_ level and a paravertebral abscess on the same level. It is known that SAFS can occur in children [3]. According to Cabet et al. the incidence of SAFS in children is much higher than the reported in literature, with half of the cases being complicated by an epidural infection [4]. The clinical presentation of SAFS in the lumbar region is similar to that of spondylodiscitis and SSEA, where the presenting symptom is mainly lumbar back pain of a varying intensity, dependant on body movement [5].

We would like to note that contrast-enhanced MRI is able to show two patterns of contrast material accumulation, depending on the predominance of pure pus or granulation tissue [6]. The classical presentation of abscess is absorption of contrast material along the periphery, while the necrotic center does not absorb contrast, due to the lack of blood vessels, resulting in so-called ring enhancement. On the contrary, if granulation tissue predominates, contrast accumulation is more homogeneous. Distinguishing the two types of epidural involvement contributes to adequate surgical planning. In Lim and Jo's case, it was a typical abscess, and in our opinion, it would have been possible to be evacuated and drained by right hemilaminectomy.

It is not entirely clear what the cause of the infection was, and whether the microbial pathogen is one or two. In the abstract and case description, the authors state that after laminectomy and drainage of the abscess, group A streptococci were found in the intraoperative pus. The intraoperative photograph presented in fig. 3 and the discussion claims that microbiological culture found methicillin-susceptible *Staphylococcus aureus.*

Our comments in no way detract from the value of Lim and Jo's article. We fully support their conclusion that a timely diagnosis of SSEA is critical in the management of the disease, requiring physicians not to rule out this diagnosis in adolescents with spinal symptoms, even in the absence of risk factors.

## Funding

None.

## Ethical approval

Not required.

## Disclosures

The authors have no personal, financial, or institutional interest in any of the drugs, materials, or devices described in this article.

## Declaration of competing interest

None declared.
